# Primary Ureteral Stump Carcinoma: Rare Presentation and Management

**DOI:** 10.7759/cureus.29103

**Published:** 2022-09-13

**Authors:** Kyler W Perry, Zachariah Taylor, Javier Piraino, Gregory McMahon

**Affiliations:** 1 General Surgery, Medical University of South Carolina, Charleston, USA; 2 Urology, Main Line Health, Wynnewood, USA; 3 Urology, Midlantic Urology, Philadelphia, USA

**Keywords:** radical nephrectomy, colon adenocarcinoma, synchronous malignancies, renal cell carcinoma, ureteral stump carcinoma

## Abstract

Primary ureteral stump carcinoma is a rare occurrence in patients who receive radical nephrectomy for renal cell carcinoma (RCC). Only 11 previous cases have been reported in the literature. We report a case of synchronous bilateral RCC and colon adenocarcinoma with the subsequent development of primary ureteral stump carcinoma that was treated with robotic ureterectomy and bladder cuff excision. To our knowledge, this is the first reported case of this presentation.

## Introduction

Primary ureteral stump carcinoma is a rare occurrence [1] that is defined as a tumor of the remaining ureteral stump after nephrectomy for reasons other than a urothelial tumor [2]. Eleven previous cases in the literature report the development of primary ureteral stump carcinoma in patients who have received a radical nephrectomy for renal cell carcinoma (RCC). Of these reports, one presented with concomitant colon adenocarcinoma and unilateral RCC [3]. We highlight a case of right ureteral stump urothelial carcinoma that developed 20 months after surgical management of synchronous bilateral RCC and right colon adenocarcinoma.

## Case presentation

**Figure 1:**
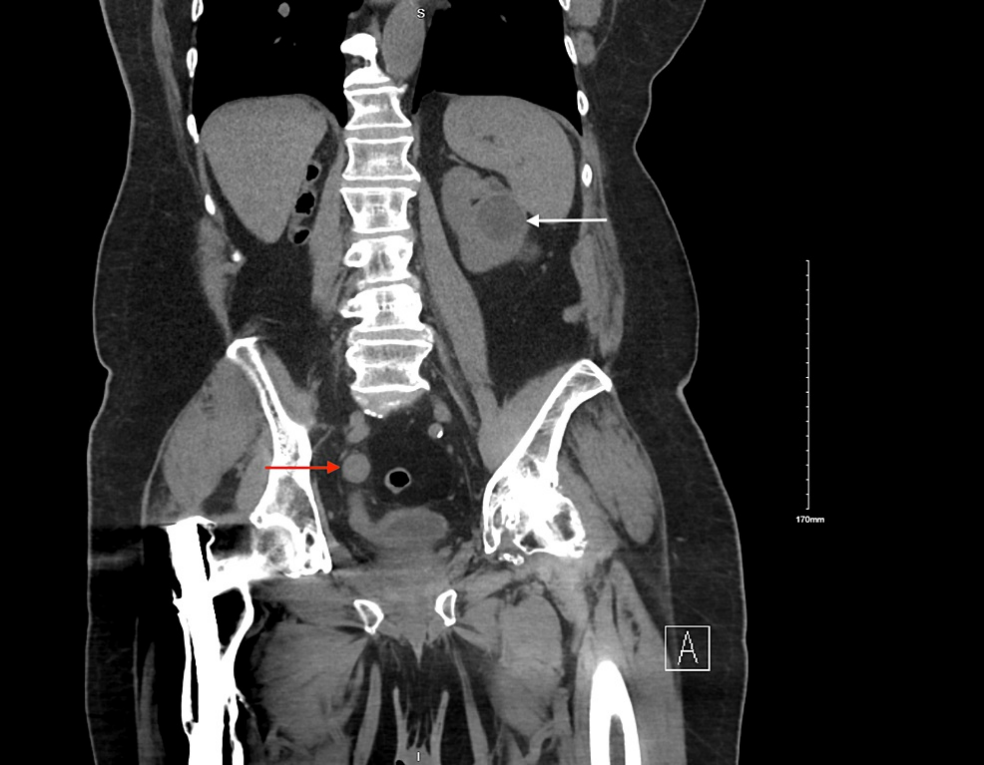
Abdominal and pelvic computed tomography (CT) showing right sided dilated ureter (red arrow) and a stable, left sided renal mass (white arrow).

A 74-year-old male presented to the emergency department with complaints of asymptomatic gross hematuria for two days. Family history was negative for genitourinary or colon cancers. The previous history was notable for bilateral RCC treated with right radical nephrectomy and left percutaneous cryoablation and colon adenocarcinoma treated with right hemicolectomy. Pathology was significant for papillary renal cell carcinoma type I of the right kidney, Fuhrman grade 2, pT2N0, with negative ureteral margins and grade 2 adenocarcinoma of the colon with mucinous features, pT3N0, with MLH1/PMS2 deletion 4. Initial creatinine was 1.7 mg/dL, elevated from a baseline of 1.0-1.4 mg/dL. The patient denied any irritative urinary symptoms such as dysuria, frequency, or urgency. The physical examination was negative for costovertebral tenderness bilaterally, abdominal tenderness, or suprapubic tenderness. Urine cytology was negative for high-grade urothelial cell carcinoma. Abdominal and pelvic computed tomography (CT) revealed stable left renal masses after treatment with percutaneous cryoablation with a dilated right ureter and adjacent mild inflammatory stranding (Figure 1). 

**Figure 2:**
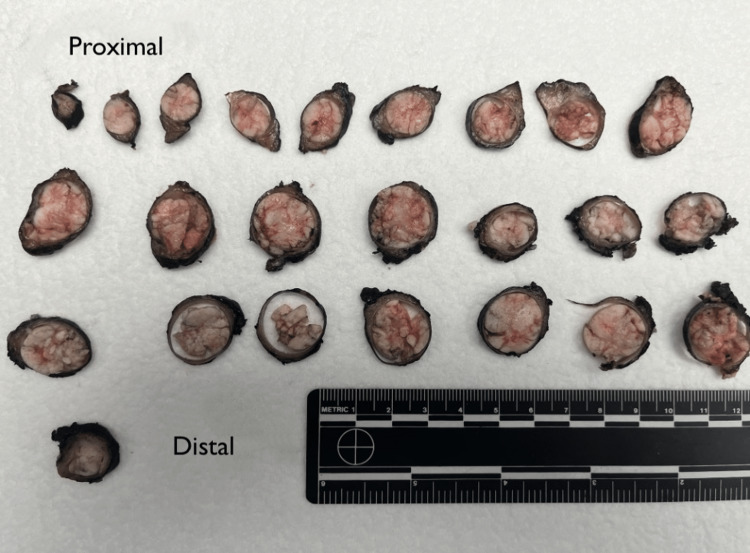
Cross-sections of the right ureter obtained from the patient, arranged from proximal (top left) to distal (bottom right).

The patient was taken to the operating room for cystoscopy and evaluation of the right ureteral stump which demonstrated a large, lumen-occupying papillary tumor within the right distal ureter and a filling defect that spanned a 4 cm segment of the right distal ureter. Operative urine cytology was positive for atypical urothelial cells, worrisome for low grade papillary urothelial neoplasm. A right robotic ureterectomy with bladder cuff excision and intravesical gemcitabine was performed. Pathology of the right ureter demonstrated low grade papillary urothelial carcinoma invading the lamina propria, pT1N0 (Figure 2). Prior to discharge, his creatinine was 1.9-2.1 mg/dL and creatinine body fluid was 2.4 mg/dL. 

## Discussion

Upper tract urothelial cancers (UTUC) are rare neoplastic growths that span from the renal calyces to the distal ureter and make up 5-10% of all urothelial tumors [4]. Primary ureteral carcinomas are rare with an overall reported incidence rate of 0.01-0.08% [1]. Primary ureteral stump carcinomas are tumors of the ureteral stump after nephrectomy for reasons other than urothelial cancer, such as benign pathology or RCC [2]. The incidence rate of primary ureteral stump carcinoma after nephrectomy for benign renal disease is 2.5% [5]. For those who have received surgical management for RCC, primary ureteral stump carcinoma is an even rarer occurrence with eleven previous reported cases in the literature. Only one case had concomitant colon adenocarcinoma, and none of the cases had bilateral synchronous RCC [3]. 

Of the previous eleven reports of ureteral stump carcinoma, all were histologically positive for urothelial carcinoma, including the present case [3,6]. Sixty-four percent of patients had an initial complaint of gross hematuria, which developed anywhere from 2 to 23 years later [3,6]. This case has the earliest reported development of primary ureteral stump carcinoma after RCC, occurring 20 months after treatment with the presentation of gross hematuria. This is the first case to report bilateral RCC before the development of primary ureteral stump malignancy. The right kidney was treated with radical nephrectomy and the left with cryoablation, and thus, only the right side had the specific environment to develop ureteral stump carcinoma. 

The development of ureteral stump carcinoma is multifactorial. One potential cause is an inflammatory environment due to infection or urinary calculi that leads to hyperplasia and metaplasia [7]. Analgesic abuse, environmental exposure, and Lynch syndrome are other known risk factors for the development of UTUC [8-10]. It is unclear if the patient had a history of analgesic abuse or known environmental risk factors. Lynch syndrome is a hereditary form of colon cancer that is an underappreciated cause of upper tract urothelial carcinomas [11]. The tumors typically develop from a germline mutation in MLH1, MSH2, MSH6, or PMS2 and have a characteristic presentation: poorly differentiated medullary-type carcinoma, mucinous adenocarcinoma, or signet-ring cells [12]. While our patient did have mucinous type colon adenocarcinoma, the genetic screening was negative for Lynch syndrome. All these factors should be considered and thought given to ureteral stump carcinoma, in patients who develop gross hematuria in the setting of multiple urologic malignancies and previous nephrectomy. 

Previous studies have highlighted the use of CT or magnetic resonance imaging (MRI) with endoscopy to diagnose ureteral stump carcinoma [13]. The ureteral stump should be monitored for the development of malignancy on surveillance CT screening in those patients who have had nephrectomy for RCC. The standard treatment for ureteral stump malignancy is total ureterectomy with bladder cuff excision [14]. This is the second reported case to use pure laparoscopy to surgically manage ureteral stump malignancy [6], with our patient receiving a robotic ureterectomy with bladder cuff excision. In these patients, particularly those with multiple malignancies, initial surgical planning may consider the removal of the ureter with bladder cuff excision to prevent the development of ureteral stump carcinoma. 

## Conclusions

In summary, the development of primary ureteral stump carcinoma after RCC is a rare occurrence. With the increasing development of ureteral cancers, and with the increased ability for surveillance screening, malignancy of the ureteral stump should be considered in any patient who develops hematuria after a nephrectomy. Treatment should consist of appropriate surgical management, which involves a ureterectomy with bladder cuff excision. 
